# The Adipovascular Unit in Lipedema: A Framework for Regional Adipose Tissue Dysregulation

**DOI:** 10.3390/cells15191820

**Published:** 2026-10-07

**Authors:** Jörg Faulhaber, C. William Hanke, Martin Barsch, Matthias Sandhofer

**Affiliations:** 1 Clinic for Dermatology, Venerology, and Allergology, University Medical Center Mannheim, Ruprecht-Karls-University Heidelberg, Theodor-Kutzer-Ufer 1-3, 68167 Mannheim, Germany; 2 Laser and Skin Surgery Center of Indiana, Indianapolis, IN 46260, USA; cwmhanke@thelassi.com; 3 Lipedema Center Linz, Starhembergstrasse 12/3, 4020 Linz, Austria; info@drbarsch.at; 4 Lipedema Center Vienna, Schönbrunnerstrasse 153/6/21, 1120 Vienna, Austria; dr.matthias@sandhofer.at

**Keywords:** lipedema, adipovascular unit, adipose stem and progenitor cells, adipose regeneration, estrogen, microvascular niche, regional susceptibility, extracellular vesicles, regenerative lock-in

## Abstract

**Highlights:**

**What are the main findings?**

- The adipovascular unit (AVU) is proposed as a system-level framework linking progenitor, vascular, immune, extracellular matrix, and adipocyte compartments in lipedema.
- The framework integrates regional susceptibility and reciprocal multicellular signaling while distinguishing lipedema-specific evidence from mechanisms inferred from general adipose-tissue biology.

**What are the implications of the main findings?**

- The AVU model generates specific, falsifiable predictions that can be tested using spatial, single-cell, multicellular, and extracellular-vesicle approaches.
- The framework distinguishes regional lipedema biology from generic obesity-associated adipose dysfunction without proposing a new anatomical structure or an established causal lesion.

**Abstract:**

Lipedema is a chronic, predominantly female disorder characterized by disproportionate regional expansion of subcutaneous adipose tissue, pain, tenderness, and easy bruising. Reported abnormalities involve adipocytes, stromal/progenitor cells, microvascular and immune compartments, extracellular matrix (ECM), and, variably, lymphatic function, but their causal and temporal relationships remain unresolved. Here, we propose the adipovascular unit (AVU) as a systems-level framework that links these compartments within a spatially organized regenerative niche. The conceptual advance is not the identification of a new anatomical structure, but the prediction that regional disease expression depends on reciprocal interactions among adipose stem and progenitor cells (ASPCs), endothelial and mural cells, immune populations, ECM, and mature adipocytes, rather than on a universal defect in any single cell type. We hypothesize that physiological female reproductive signals may expose a regionally susceptible AVU in which regenerative responses are inadequately constrained in space, magnitude, or duration. This framework also distinguishes lipedema from obesity-associated adipose dysfunction: lipedema is regionally patterned and may coexist with relatively preserved systemic metabolic function, whereas obesity-related adipose dysfunction is more closely linked to whole-body energy excess, ectopic fat, insulin resistance and cardiometabolic risk. Evidence demonstrated directly in lipedema is separated throughout from general adipose-tissue biology used to establish plausibility and from mechanisms that remain hypothetical. We derive falsifiable predictions involving depot-specific molecular states, endocrine context, multicellular cross-talk, extracellular-vesicle cargo and transfer, spatial biological boundaries, and persistence after stimulus withdrawal. Testing these predictions in paired-depot, spatially resolved and multicellular systems will determine whether AVU dysregulation is a causal organizing principle of lipedema or a descriptive consequence of established disease.

## 1. Introduction

Lipedema is a chronic disorder of subcutaneous adipose tissue characterized by disproportionate, typically symmetrical extremity enlargement, pain and tenderness, easy bruising, and a striking female predominance. Histological and molecular studies report adipocyte hypertrophy, altered adipogenic differentiation, microvascular abnormalities, extracellular matrix (ECM) remodeling, immune-cell changes, and variable lymphatic dysfunction, but their temporal and causal relationships remain unresolved [1,2,3].

Its association with puberty, pregnancy, and menopause implicates female reproductive physiology without establishing systemic hormone excess as the cause. A more plausible possibility is that physiological endocrine signals act on regionally susceptible adipose tissue. This is biologically credible because adult adipose tissue undergoes continuous cellular turnover [4], while adipose stem/progenitor and microvascular compartments form spatially organized regenerative microenvironments [5]. Sex- and estrogen-dependent adipose biology provides additional context for depot-specific responses [6,7,8].

Lipedema-specific studies report altered stromal/progenitor phenotypes and adipogenic responses [9,10,11,12], characteristic extracellular-vesicle (EV) cargo [13], and vascular or immune abnormalities [14,15,16]. Results vary with anatomical sampling and experimental context. Such heterogeneity argues against a simple cell-autonomous hyperadipogenic disorder and instead suggests dysregulation of a multicellular regenerative system.

We therefore propose the adipovascular unit (AVU) as a spatially organized regenerative niche integrating ASPCs, microvascular endothelial and mural cells, immune populations, ECM, mature adipocytes, and their paracrine and EV-mediated communication. The mechanistic advantage of the AVU framework is that it makes regionality and cross-compartment dependence explicit: it predicts that a disease-associated phenotype should emerge from the state and interactions of the local niche, not simply from increased adipocyte mass or an isolated progenitor abnormality. This distinguishes the model from conventional obesity-associated adipose dysfunction, in which systemic energy surplus, adipose expandability, ectopic lipid deposition, and metabolic impairment are central organizing features [17]. We hypothesize that lipedema may arise when physiological female hormone-responsive remodeling encounters a regionally susceptible AVU in which regenerative activity is inadequately constrained in space, magnitude, or duration. Reciprocal cross-compartment remodeling could then amplify and stabilize the altered tissue state. The following sections distinguish lipedema-specific observations from general adipose-biology evidence and from testable hypotheses derived from the model.

## 2. The Adipovascular Unit as a Regenerative Niche

Adipose tissue remodels throughout adult life through adipocyte hypertrophy and, when required, hyperplasia driven by resident ASPCs. Progenitor identity and differentiation are influenced by sex, anatomical location, and the local microvascular environment [4,5]. Single-cell and spatial studies further show that adipose stem cells are heterogeneous fibroblast-like populations that contribute both adipocyte precursors and ECM-regulating functions, with sexually dimorphic transcriptional states and adipogenic commitment linked to position within the microvascular niche [5]. A single-cell atlas of human and mouse white adipose tissue maps depot-associated cellular heterogeneity and intercellular organization [18], while a mesenchymal progenitor hierarchy provides further context for adipogenic niche regulation [19].

Recent single-cell mapping of the human subcutaneous adipose vascular niche further demonstrates substantial endothelial heterogeneity and phenotypic plasticity. AlZaim et al. identified canonical arterial, venous, capillary, and lymphatic endothelial populations together with a heterogeneous sub-endothelial population exhibiting mixed endothelial, mesenchymal, adipogenic, and immune transcriptional features [20]. Computational trajectory analyses and experimental validation suggested that some of these states may emerge through partial endothelial-to-mesenchymal transition (EndMT), highlighting a previously underappreciated plasticity of the adipose vascular compartment. Importantly, these observations were obtained in adipose tissue across metabolic states rather than in lipedema and therefore provide biological plausibility for an integrated adipovascular niche without demonstrating disease-specific EndMT in lipedema.

A key distinction is that ASPCs and vascular mural cells are closely associated but biologically distinguishable populations [5]. The microvasculature is therefore not simply a supply network: endothelial cells, mural cells, progenitors, immune cells, stromal cells, and ECM form a spatially coupled environment in which reciprocal signaling coordinates tissue expansion. Regional differences in progenitor biology and adipose function provide a plausible substrate for the characteristic distribution of lipedema despite uniform systemic endocrine exposure [5,7,8].

### 2.1. Defining the Adipovascular Unit

**Figure 1:**
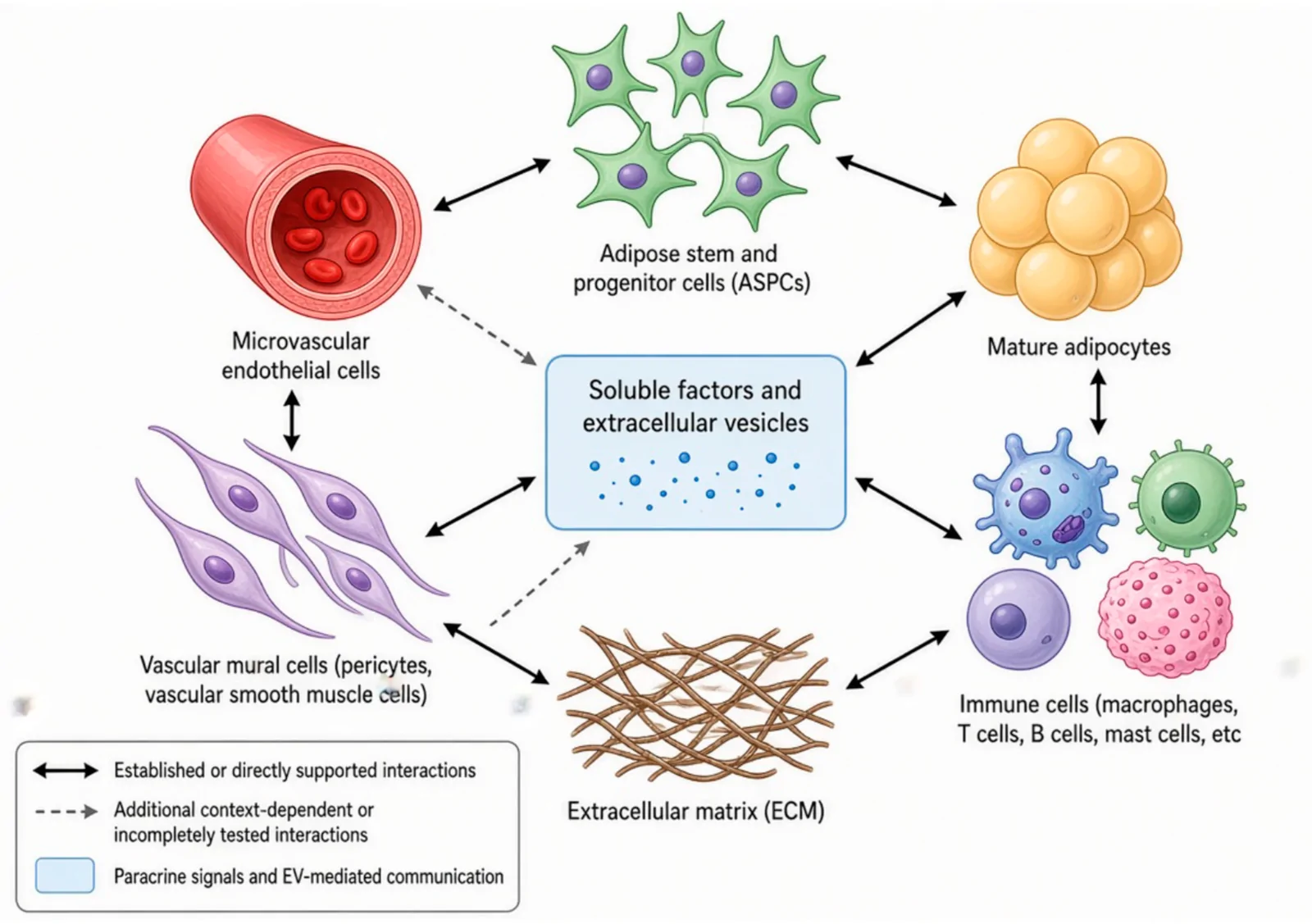
The adipovascular unit in lipedema: multicellular architecture and reciprocal communication. The adipovascular unit (AVU) is proposed as a spatially organized regenerative niche of subcutaneous adipose tissue comprising adipose stem and progenitor cells (ASPCs), microvascular endothelial cells, mural cells, mature adipocytes, immune populations, and extracellular matrix. These compartments interact through direct cell–cell contacts and paracrine communication, including soluble mediators and extracellular vesicles; solid arrows indicate established or directly supported interactions, whereas dashed arrows denote additional context-dependent or incompletely tested relationships. The figure is intended to emphasize the AVU as a reciprocal multicellular network rather than a linear sequence and does not imply that a new anatomically discrete structure has been demonstrated.

We define the adipovascular unit (AVU) as a functional and spatial framework describing the regenerative microenvironment in which adipose progenitor biology and microvascular adaptation are coordinated (Figure 1). Its principal components are ASPCs; microvascular endothelial cells; vascular mural cells, including pericytes and vascular smooth muscle cells; ECM; resident and recruited immune cells; and mature adipocytes. These components influence one another through direct cellular interactions, soluble mediators, matrix-dependent signaling, and EV-mediated communication.

The AVU is not proposed as a newly discovered anatomical organ but as a systems-level construct for a spatially coupled regenerative niche. Here, ‘regenerative niche’ refers to the local cellular and extracellular environment that regulates progenitor recruitment and differentiation while coordinating the vascular, immune, and matrix adaptation required for adipose-tissue remodeling. Reciprocity is central: adipose expansion requires vascular and matrix adaptation, whereas endothelial, mural, immune and matrix states can alter progenitor behavior and adipocyte function. Applied to lipedema, the mechanistic proposition is that failure of this coordination—rather than abnormality of a single compartment—can generate a regionally persistent tissue phenotype. This architecture updates our earlier predominantly sequential regenerative-imbalance model toward a network model in which no single compartment is assumed a priori to be the universal initiating lesion [21]. Figure 1 is organized accordingly: it places the principal cellular and matrix compartments around shared paracrine/EV-mediated communication and uses bidirectional interactions to emphasize reciprocal regulation rather than a linear causal sequence.

### 2.2. Physiological Adipose Regeneration and Regional Susceptibility

Hormone-responsive adipose regeneration is physiological. Female subcutaneous adipose tissue has greater capacity for hyperplastic expansion, and estrogen can preserve or recruit committed progenitor populations [6,7,8]. The relevant distinction is therefore not the presence of estrogen-responsive adipogenesis, but whether its regional control, magnitude, duration, or termination is altered.

Three observations motivate this interpretation. First, affected and spared depots coexist within the same systemic endocrine environment, arguing against a purely systemic explanation. Second, normal adipose progenitor biology is sexually dimorphic and estrogen responsive [5,6], and sex-dependent adipose distribution and estrogen-receptor signaling are well established in general adipose biology [7,8]. Third, lipedema-specific studies report abnormalities in stromal/progenitor behavior [9,10,11,12], EV miRNA cargo [13], vascular/perivascular biology [14,16], immune signaling [15], and stage-associated adipocyte hypertrophy and fibrosis [22]. Lymphatic and interstitial abnormalities are heterogeneous and are considered separately below [23,24,25,26,27,28,29,30]. These observations do not establish a common cause, but they are compatible with dysregulation of a shared regenerative tissue system rather than a set of unrelated lesions.

### 2.3. From Physiological to Dysregulated AVU

A useful distinction can therefore be drawn between physiological and dysregulated AVU states. In physiological remodeling, endocrine and metabolic signals induce controlled ASPC recruitment, coordinated microvascular and ECM adaptation, limited adipocyte formation, and restoration of tissue homeostasis. In the proposed lipedema state, the same class of physiological signals encounters a regionally susceptible AVU, producing altered progenitor behavior together with vascular, stromal, immune, and matrix remodeling and disproportionate adipose expansion.

This sequence is a hypothesis, not an experimentally established pathway. It predicts that affected and spared depots should differ in progenitor state, cellular composition, spatial organization, or response to standardized endocrine stimulation; that perturbing one AVU compartment should influence others if cross-talk is causal; and that a self-reinforcing state, if present, should show at least partial persistence after removal of the initiating stimulus.

## 3. Female Reproductive Physiology and Regional AVU Susceptibility

The striking female predominance of lipedema and its association with reproductive transitions require a sex-specific explanation, but they do not demonstrate pathological circulating estrogen concentrations. Three levels of biology may intersect: sex-dependent adipose progenitor programs, reproductive-hormone responsiveness, and inherited or developmental susceptibility. Familial clustering and genetic association support a heritable component, although no single causative gene explains the disorder [31,32]. The central AVU question is therefore whether sex-limited or female-enriched susceptibility is expressed locally when physiological reproductive signals act on anatomically distinct adipose niches.

### 3.1. Sexual Dimorphism and Hormone-Responsive Adipose Regeneration

Women preferentially accumulate subcutaneous adipose tissue in gluteofemoral and other peripheral depots, reflecting sex- and depot-dependent adipose biology rather than differences in mature adipocyte size alone [7,8]. Scheidl et al. showed in mice that female subcutaneous depots preserve and recruit committed progenitors more effectively during adipogenic stimulation; ovariectomy reduced this phenotype and 17β-estradiol restored it [6]. Estrogen-responsive hyperplastic expansion is therefore part of normal female adipose physiology.

Uhrbom et al. independently demonstrated sexual dimorphism in adipose stem-cell transcriptional programs and showed that adipogenic commitment is related to anatomical position within the microvascular niche [5]. Human adipose distribution is also strongly sex- and depot-dependent [7], while estrogen-receptor signaling can modulate adipose expansion, inflammation, and fibrosis [8]. In lipedema-derived cells, estrogen exposure can alter adipogenic-marker expression in vitro [12]. These observations provide a mechanistic bridge between female predominance and regional distribution, but they do not prove that estrogen initiates lipedema. Within the AVU framework, genetic/developmental susceptibility could set a depot-specific response threshold, while physiological reproductive signals reveal that susceptibility through effects on progenitor, vascular, stromal, and matrix compartments.

### 3.2. Reproductive Transitions and Altered Estrogen Responsiveness in Lipedema

Clinical descriptions associate lipedema onset or progression with puberty, pregnancy, and menopause, but these transitions represent very different endocrine states [2,33]. Their common feature is not a uniform estrogen concentration but the need for substantial adaptation to changing reproductive, metabolic, and vascular demands. Within the AVU model, reproductive transitions are therefore viewed as physiological challenges to regional regenerative systems rather than episodes of simple estrogen excess.

Evidence must be separated by level. In general adipose biology, estrogen regulates the female adipose progenitor compartment in vivo [6]. In lipedema-specific in vitro models, 17beta-estradiol has produced context-dependent changes in estrogen-receptor and adipogenic signaling [11,12]. These findings support biological plausibility for altered endocrine responsiveness but do not establish an in vivo hormonal driver of lipedema. The dependence of observed responses on experimental dimensionality further argues that cell–cell, cell–matrix, and niche interactions should be tested rather than inferred from isolated cells alone.

Adipose tissue is capable of local estrogen production through aromatase-dependent steroid metabolism [34,35,36]. In lipedema-specific work, Strohmeier et al. reported increased CYP19A1 expression in affected thigh adipose tissue and increased ZNF423 expression in stromal vascular fraction, endothelial and pericyte compartments [14]. These findings are compatible with altered local estrogen metabolism and adipogenic signaling within the vascular niche, but they do not demonstrate increased aromatase activity, elevated local estradiol concentrations, or an initiating role for aromatase. The relevant hypothesis is therefore altered local processing and response to physiological endocrine signals, not systemic estrogen excess.

### 3.3. The Regionality Paradox and Regional AVU Susceptibility

A purely endocrine explanation faces a fundamental regionality problem: puberty, pregnancy, and menopause alter the endocrine environment of the entire organism, whereas lipedema selectively affects characteristic extremity depots while sparing others. If the signal is systemic, why is the pathological response regional?

We propose regional AVU susceptibility as the missing variable. Depot-specific developmental programs, progenitor composition, vascular organization, ECM mechanics, local steroid metabolism, immune tone, and lymphatic/interstitial capacity could create different response thresholds among adipose regions [5,7,8,14]. This does not identify which feature is primary; it predicts that susceptibility should be measurable by comparing affected and spared depots within the same individual.

### 3.4. From Physiological Adaptation to Pathological Persistence

The transition from physiological adaptation to disease may therefore depend on failure to terminate or spatially constrain a normally adaptive regenerative response. Reproductive or metabolic signals could transiently increase progenitor recruitment, vascular adaptation, and matrix remodeling; in a susceptible AVU, reciprocal interactions among these compartments could prolong or amplify the response after the original stimulus has diminished.

This proposed persistence is termed regenerative lock-in. It is not established by current evidence and should not be interpreted as irreversible disease or as proof that a particular compartment initiates lipedema. Rather, it is a testable systems-level possibility: disease-associated cellular behavior should persist at least partly after withdrawal of candidate stimuli and should depend on multicellular context if reciprocal reinforcement is mechanistically important.

## 4. From Regional Susceptibility to Regenerative Imbalance and Multicellular Amplification

**Figure 2:**
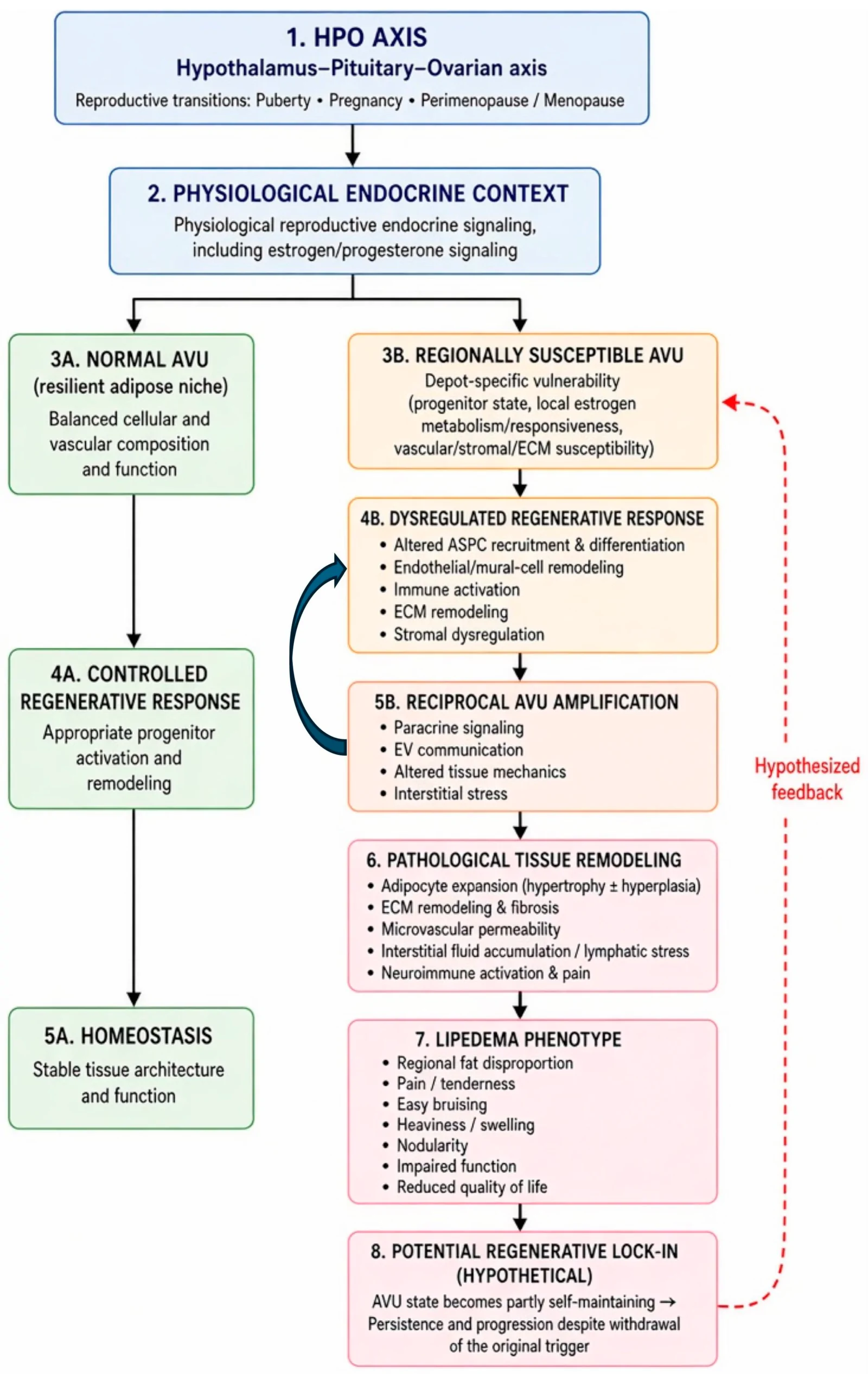
From physiological endocrine context to regional AVU dysregulation and the lipedema phenotype: a working model. The hypothalamic–pituitary–ovarian axis provides a physiological reproductive endocrine context across puberty, pregnancy, perimenopause and menopause. In a resilient AVU, these signals are accommodated by controlled regenerative responses and tissue homeostasis; in a regionally susceptible AVU, the same physiological context may encounter a lower threshold for regenerative activation, leading to dysregulated progenitor recruitment and differentiation, endothelial–stromal and immune cross-talk, extracellular matrix remodeling and reciprocal AVU amplification. Progressive tissue remodeling may contribute to the characteristic regional adipose expansion, pain, tenderness, easy bruising, heaviness or swelling, and nodularity or fibrosis. Persistence of an altered AVU state after withdrawal of a candidate initiating or amplifying stimulus is shown as a hypothetical regenerative lock-in and remains to be experimentally demonstrated. The downward arrangement is a schematic entry-to-phenotype overview rather than a linear causal chain: reciprocal interactions occur within the AVU amplification node, and the dashed return arrow denotes the hypothesized feedback from regenerative lock-in to regional AVU susceptibility.

If regional susceptibility determines where lipedema develops, the next question is how a local regenerative response could become a stable multicellular tissue phenotype. Lipedema-specific studies report altered stromal/progenitor or adipogenic phenotypes [9,10,11,12], EV cargo [13], vascular/perivascular changes [14,16], macrophage-associated effects on adipocyte differentiation [15], and stage-associated adipocyte and ECM remodeling [22]. Pathway-level and multi-omics analyses additionally identify broader molecular abnormalities [37,38]. None of these observations establishes a temporal hierarchy. General adipose-tissue studies provide mechanistic examples of reciprocal vascular–stromal signaling [5,20]; these are used here to formulate testable mechanisms, not as evidence that the same pathways are already demonstrated in lipedema. Figure 2 summarizes this evidence-to-hypothesis transition from physiological endocrine context through regional susceptibility and multicellular amplification to the clinical phenotype.

### 4.1. Regenerative Imbalance and Altered Progenitor Control

Lipedema tissue shows altered stromal/progenitor phenotypes and adipogenic responses, but the direction and magnitude of these changes vary across experimental systems [9,10,11,12]. This variability argues against a universal intrinsic hyperadipogenic defect. Instead, progenitor behavior may depend on disease stage, anatomical site, hormonal context, and the cellular and matrix environment in which ASPCs are studied.

Within the AVU model, regenerative imbalance therefore means loss of coordinated control rather than simply increased adipogenesis: progenitor recruitment, differentiation, and termination become mismatched to vascular, matrix, and tissue demands.

### 4.2. Multicellular Communication Within the Dysregulated AVU

Evidence for cross-compartment communication is emerging. Lipedema-derived SVF EVs carry characteristic miRNA cargo [13]; endothelial and perivascular abnormalities occur in early-stage tissue [14]; and macrophage-associated signals can influence adipocyte differentiation [15]. These findings demonstrate potential communication routes but do not establish a single causal direction.

The AVU model therefore treats endothelial, mural, progenitor, immune, and matrix compartments as a coupled network. This network interpretation is supported by recent single-cell connectomic analyses of human subcutaneous adipose tissue, in which adipocytes, adipose stem and progenitor cells, and adipose endothelial populations emerged as major participants in metabolic-state-dependent intercellular communication. Altered VEGF, TGF-β, and BMP signaling, together with collagen- and laminin-associated communication involving endothelial subpopulations, further illustrates how vascular, stromal, and adipocyte compartments can behave as an integrated signaling system. Whether analogous communication networks are altered in lipedema remains to be determined [20]. Functional co-culture, transfer, and perturbation experiments are required to determine which abnormalities are transferable, necessary, or sufficient and whether the strongest disease phenotype emerges only in a multicellular context.

### 4.3. Adipocyte Expansion, Extracellular-Matrix Remodeling, and Interstitial Homeostasis

Established lipedema tissue shows adipocyte hypertrophy, adipose remodeling, and stage-associated fibrosis [16,22,39]. ECM changes may alter tissue mechanics and progenitor behavior, while adipocyte expansion increases demands on perfusion, interstitial clearance, and structural support. These relationships are compatible with reciprocal amplification but do not establish whether ECM remodeling is initiating, adaptive, or secondary.

Interstitial and lymphatic findings are similarly heterogeneous. Increased tissue fluid and functional or morphological lymphatic abnormalities have been reported, but primary universal lymphatic failure has not been demonstrated [23,24,25,26,27,28,29,30]. The AVU model therefore positions interstitial and lymphatic dysfunction as possible modifiers, amplifiers, or consequences whose role may vary by phenotype and disease stage.

### 4.4. Multicellular Amplification and Regenerative Lock-In

A central hypothesis is that reciprocal remodeling can stabilize an abnormal regional state. Lipedema studies provide plausible components of such a network, including vascular/perivascular abnormalities [14,16], adipocyte death and regeneration [39], macrophage-associated effects on adipocyte differentiation [15], broader molecular pathway alterations [38], and stage-associated inflammation and fibrosis [22]. These findings can support candidate interactions, but no current study demonstrates the complete reciprocal loop or its temporal sequence.

We use regenerative lock-in to describe a specific, testable possibility: reciprocal cellular signaling maintains a disease-associated AVU state for a measurable period after withdrawal of an initiating or amplifying stimulus. This is conceptually narrower than fibrosis or structural stabilization in chronic remodeling. Fibrosis can preserve altered tissue architecture without demonstrating an actively self-maintaining multicellular signaling network. Regenerative lock-in would therefore require functional evidence of persistence in patient-derived multicellular systems and, ideally, reversal after targeted disruption of a causal network node; rapid normalization after stimulus withdrawal would argue against it. No lipedema-specific study has yet demonstrated this phenomenon.

### 4.5. From Dynamic Dysregulation to a Self-Reinforcing Adipovascular System

The proposed disease process is therefore better represented as a dynamic network than a linear cascade. Different upstream susceptibilities may enter through progenitor, vascular, immune, matrix, endocrine, or lymphatic pathways and converge on a regional AVU state. Figure 2 translates the text of Section 3 and Section 4 into a working model: the left branch represents controlled physiological remodeling, whereas the right branch depicts the hypothesized sequence from regional susceptibility to dysregulated regenerative response, reciprocal amplification, and tissue remodeling. The dashed feedback arrow is deliberately restricted to the unproven regenerative lock-in hypothesis. The model does not require the same initiating lesion in every patient; its central claim is that regional multicellular interactions may organize and, if experimentally confirmed, help maintain the phenotype. The directional layout summarizes the main conceptual progression, not a unidirectional mechanism; the reciprocal amplification step and dashed feedback represent network interactions and the separately hypothetical persistence loop.

## 5. From AVU Dysregulation to the Clinical Lipedema Phenotype

A mechanistic model of lipedema is only meaningful if it can account for the established clinical phenotype without replacing clinical classification. Lipedema is defined by a characteristic combination of disproportionate regional adipose-tissue expansion and symptoms rather than excess adipose mass alone. Current clinical descriptions classify patients by anatomical distribution and by morphological stage; these descriptive categories should remain distinct from any proposed molecular AVU state [2,3]. Common diagnostic features include pain or pressure sensitivity, bilateral and symmetrical disproportionate enlargement of the extremities, easy bruising, a negative Stemmer sign, and characteristic sparing of the feet [1,2,3,40]. We therefore anchor the AVU discussion below to four established clinical dimensions—distribution, tissue morphology/stage, vascular-interstitial manifestations and pain/function—while avoiding the unsupported assumption that each clinical type has a unique molecular mechanism.

### 5.1. Anatomical Distribution, Stage and Structural Remodeling

The established distribution-based clinical types describe where disproportionate adipose tissue is expressed, whereas clinical stages describe surface and tissue morphology [2,3]. The AVU framework does not redefine these categories. Instead, it predicts that the anatomical pattern should correspond to depot-specific biological susceptibility and that progressive structural remodeling may contribute to stage-related morphology. The characteristic bilateral and symmetrical enlargement of affected limbs with frequent sparing of the feet [1,2,3] is compatible with depot-specific susceptibility, but this interpretation remains hypothetical until affected-spared boundaries are mapped biologically.

Histological studies demonstrate adipocyte enlargement in established lipedema [16,22], whereas experimental studies indicate altered or context-dependent adipogenic differentiation potential [9,10,11,12]. The AVU model therefore considers adipocyte hypertrophy and potentially altered hyperplastic recruitment as complementary contributors to tissue expansion. Their relative contributions may vary between patients and disease stages. Increasing interstitial collagen and stage-associated fibrosis further suggest that progressive matrix remodeling may convert a predominantly dynamic cellular abnormality into an increasingly structural tissue state [22].

### 5.2. Microvascular and Interstitial Manifestations

Easy bruising is characteristic of lipedema, and clinical and experimental studies describe capillary fragility, vascular dilation, increased permeability, and altered endothelial behavior [1,14,16]. Because bruising also depends on vessel-wall integrity, mechanical trauma, perivascular support, and coagulation, its precise structural basis remains unresolved. It is nevertheless compatible with altered microvascular and perivascular stability within the affected AVU.

Heaviness and swelling likewise need not imply primary lymphatic failure. Lipedema should not be equated with primary lymphedema, and imaging studies demonstrate heterogeneous lymphatic findings ranging from largely preserved transport to subclinical abnormalities in subsets of patients [23,24,25,26,27,28,29,30]. Disturbed interstitial homeostasis and an emerging load–clearance mismatch could therefore contribute to symptoms before or without severe lymphatic dysfunction.

### 5.3. Pain and the Dissociation Between Symptoms and Morphology

Pain and pressure sensitivity are defining features of lipedema. Studies support peripheral pain, dermal hypersensitivity, and neurogenic inflammatory signaling, while quantitative sensory testing demonstrates regionally altered sensory thresholds [41,42,43]. Potential contributors include altered interstitial mechanics, fibrosis, vascular dysfunction, inflammatory mediators, tissue pressure and peripheral sensory dysfunction, but their causal hierarchy remains unresolved.

Pain may therefore represent an emergent neurovascular–stromal phenotype rather than the endpoint of a simple linear pathway. Morphological stage and symptom severity are not necessarily equivalent [22,43], implying that patients with similar external morphology may occupy different underlying biological states.

### 5.4. Persistence and Progression as Loss of Tissue Homeostasis

If reproductive transitions contribute to disease manifestation, persistence after the initiating transition has resolved implies durable structural consequences, continuing tissue-level maintenance, or both. The regenerative lock-in hypothesis captures the latter possibility, although clinical persistence alone does not establish it.

Biological progression may involve a shift from altered cellular signaling and regenerative responsiveness toward adipose expansion, multicellular amplification, fibrosis, altered interstitial mechanics, and potentially lymphatic impairment [22]. This sequence remains hypothetical and should not replace established clinical staging; rather, it predicts that the dominant biological mechanisms may change during disease progression.

### 5.5. A Convergent Clinical Phenotype

Regional adipose expansion, bruising, heaviness and swelling, fibrotic remodeling and pain may thus represent different macroscopic manifestations of disturbed regional tissue homeostasis rather than independent pathological processes. The characteristic lipedema phenotype could consequently reflect a regionally susceptible adipose niche in which dysregulated regeneration alters adipose volume, vascular and interstitial behavior, extracellular matrix architecture and sensory function.

This interpretation generates experimentally testable relationships: affected and spared depots within the same woman should differ biologically; local vascular abnormalities should associate with regional tissue phenotype; and vascular filtration, interstitial-fluid dynamics and lymphatic transport should show phenotype- or stage-dependent relationships. These predictions connect the clinical phenotype directly to experimental validation.

### 5.6. Metabolic Health and Distinction from Obesity

Lipedema and obesity frequently coexist and share adipocyte hypertrophy, inflammation, ECM remodeling, and vascular changes, but they should not be treated as biologically interchangeable. Obesity-associated adipose dysfunction is commonly linked to limited adipose expandability, ectopic lipid deposition, insulin resistance, and systemic cardiometabolic risk [17]. In contrast, recent clinical studies suggest that women with lipedema can show greater lower-body adiposity with comparatively preserved insulin sensitivity or fewer metabolic abnormalities than women with obesity matched for BMI or adiposity [44,45]. These findings do not imply that lipedema is metabolically benign: coexisting obesity remains clinically important and can dominate metabolic risk.

The distinction is mechanistically relevant to the AVU model. A lipedema-specific AVU signature should track regional disease distribution, pain, vascular/interstitial features, or tissue remodeling beyond what is explained by BMI, total fat mass, or generic obesity-associated inflammation. Studies should therefore compare affected and spared depots within the same patient and include obesity-without-lipedema controls. If putative AVU abnormalities are fully explained by obesity or total adiposity, their specificity for lipedema would be substantially weakened.

## 6. Testable Predictions and Experimental Validation of the AVU Hypothesis

The value of the adipovascular unit (AVU) hypothesis depends on whether it generates predictions that distinguish it from alternative explanations of lipedema. If lipedema reflects persistent dysregulation of a regionally susceptible adipose-tissue niche, reproducible biological differences should exist between affected and spared depots, and disease-associated phenotypes should depend at least partly on interactions among AVU compartments. The following predictions provide a framework for experimental validation or falsification.

### 6.1. Regional and Context-Dependent Predictions

Existing paired-depot, progenitor, and hormonal-response studies demonstrate regional and context-dependent differences but do not establish whether these differences define anatomical susceptibility in lipedema [6,12,14,22].

Prediction 1—Depot-specific AVU molecular signature. Affected adipose tissue should show a reproducible multicellular signature relative to anatomically spared tissue from the same patient, for example, coordinated differences in ASPC state/adipogenic commitment, endothelial or perivascular programs, ECM/fibrotic signaling, and immune composition rather than a nonspecific increase in adipocyte size alone. Spatial or single-cell profiling should test whether such features co-segregate within affected depots after adjustment for BMI, total adiposity, and clinical stage. Repeated biological equivalence between affected and spared depots, or a signature fully explained by obesity, would substantially weaken the concept of regional AVU susceptibility.

Prediction 2—Hormonal context dependence. Physiologically relevant reproductive endocrine signals should elicit different responses in affected and spared AVUs from the same patient. Identical responses across relevant hormonal conditions would argue against differential regional endocrine responsiveness as a major determinant of susceptibility.

Prediction 3—Biological boundary. Spatially resolved sampling across the clinical transition from affected to spared tissue should reveal a corresponding molecular, cellular, structural, or functional AVU transition. Failure to identify such a boundary despite precise spatial sampling would challenge the concept of regionally encoded susceptibility.

### 6.2. Multicellular Communication and Causal Reconstruction

Available functional studies demonstrate selected cross-compartment effects, but their necessity, directionality, and sufficiency remain unresolved [13,14,15].

Prediction 4—Multicellular dependence. Multicellular systems incorporating endothelial, mural, stromal/progenitor, immune, matrix, and adipocyte compartments should reproduce disease-associated behavior more completely than isolated monocultures. Robust reproduction of the relevant phenotype by isolated cells alone would reduce the necessity of multicellular AVU dysfunction.

Prediction 5—Endothelial–stromal cross-talk and vascular plasticity. Reciprocal co-culture of endothelial and stromal/progenitor cells from affected, spared, and control tissue should determine whether abnormalities are compartment-intrinsic or emerge through interaction. Single-cell and spatial approaches should additionally test whether affected lipedema tissue contains altered endothelial states, including mesenchymal-, progenitor-, or immune-associated transcriptional programs, and whether such states differ between affected and spared depots. Absence of reproducible reciprocal effects or disease-associated endothelial-state differences would weaken a central adipovascular prediction.

Prediction 6—Extracellular-vesicle cargo and functional transfer. Lipedema-derived SVF EVs have been reported to contain a distinct miRNA profile, including differential abundance of miR-16-5p, miR-29a-3p, miR-24-3p, miR-454-3p, miR-144-5p, miR-130a-3p and let-7c-5p [13]. The AVU hypothesis predicts that one or more of these cargo differences, or subsequently validated lipedema-specific EV cargo, should alter recipient endothelial, stromal/progenitor, immune, or adipogenic phenotypes under physiologically relevant conditions. Candidate-cargo gain/loss experiments should attenuate or reproduce the transferred phenotype. Failure of reproducible transfer, or absence of cargo-dependent effects, would reduce the proposed role of EV-mediated amplification.

Prediction 7—Network perturbation. Selective perturbation or normalization of one pathological AVU compartment should produce secondary changes in other compartments. Consistent independence of individual compartments would argue against the proposed network architecture.

### 6.3. AVU States, Clinical Phenotype and Regenerative Lock-In

Disease-associated molecular signatures and stage-dependent remodeling suggest that lipedema may comprise distinct biological states, although longitudinal evidence is lacking [22,38].

Prediction 8—Changing AVU states. Reproducible AVU-state patterns or trajectories should emerge from combinations of adipogenic activity, vascular dysfunction, immune composition, matrix remodeling, interstitial disturbance, and potentially neural involvement [22]. If these abnormalities merely track tissue volume, BMI, or conventional stage, AVU-state classification would add limited biological information.

Prediction 9—Regenerative persistence. In patient-derived multicellular systems, disease-associated features should persist for a measurable period after withdrawal of a candidate initiating or amplifying stimulus if regenerative lock-in exists. Rapid and complete normalization after stimulus withdrawal would argue against a self-maintaining AVU state.

Prediction 10—Clinical correlation. AVU abnormalities should show reproducible relationships to anatomical distribution, symptoms, progression, or other clinically meaningful phenotypes. Biological abnormalities without such relationships would have uncertain relevance to lipedema pathogenesis.

### 6.4. Experimental Roadmap and Falsification

A staged experimental strategy could progress from spatial definition in paired affected and spared tissue, through multicellular reconstruction and causal perturbation, to clinical correlation and therapeutic response. The AVU hypothesis would be weakened by biological equivalence of affected and spared tissue, predominantly cell-autonomous abnormalities, absence of functional cross-compartment interactions or regional endocrine-response differences, lack of a biological correlate of anatomical distribution, or absence of meaningful clinical associations. Conversely, demonstration that regional reciprocal interactions among AVU compartments are required to reproduce disease-associated phenotypes—and that targeted disruption attenuates or reverses them—would provide strong support for the model. The principal experimental predictions, preferred approaches, and findings that would weaken the AVU model are summarized in Table 1.

## 7. Clinical and Therapeutic Implications of the AVU Model

The AVU hypothesis is a pathogenetic framework rather than a proposal for immediate changes in clinical management. Current treatment remains directed toward symptom reduction, preservation of mobility and function, management of coexisting obesity and metabolic comorbidity, and, in selected patients, reduction in pathological adipose-tissue burden. The metabolic distinction discussed above is clinically important: lipedema itself should not be equated with obesity, while obesity, when present, warrants evidence-based treatment because it can add independent metabolic and mechanical burden [2,3,44,45]. The potential clinical contribution of the AVU framework is to distinguish phenotype-directed treatment from future mechanism-directed intervention.

### 7.1. Phenotype-Directed Treatment

Compression, physical activity, weight management, rehabilitation and other conservative strategies can reduce symptoms or improve function through effects on mechanical load, interstitial fluid handling, mobility or associated metabolic factors [2,3]. Such benefits neither establish nor exclude a specific initiating mechanism and do not remove the affected adipose-tissue compartment itself.

### 7.2. Reduction Surgery and the Affected Tissue Niche

Lipedema reduction surgery occupies a distinct position because it physically removes substantial amounts of affected subcutaneous adipose tissue rather than primarily modifying downstream manifestations [46,47,48]. Liposuction removes not only adipocytes but also components of the stromal, vascular, immune, extracellular matrix, and interstitial environment.

Within the AVU framework, reduction surgery may therefore reduce pathological tissue mass and mechanical burden while simultaneously disrupting part of a multicellular niche capable of sustaining the altered tissue state. Whether this constitutes true disease modification, or whether residual susceptible tissue can reconstruct the pathological AVU state, remains unknown. Longitudinal characterization before and after surgery could test whether tissue removal produces biological changes extending beyond those attributable to reduced tissue volume alone.

Tissue-preserving surgical principles are compatible with reducing affected tissue burden while minimizing unnecessary injury to vascular, lymphatic, neural, and connective-tissue structures [49,50,51,52]. The AVU hypothesis does not establish superiority of one surgical technology over another.

### 7.3. From Phenotype-Directed to Mechanism-Directed Intervention

If causal interactions maintaining AVU dysregulation are identified, potential intervention points could include progenitor–vascular signaling, immune–stromal communication, extracellular-vesicle-mediated signaling, extracellular-matrix remodeling, or local endocrine signal processing. Candidate targets should progress from association to functional transfer, causal perturbation, and phenotypic normalization before being considered disease-modifying.

Importantly, physiological female subcutaneous adipose expansion depends on regulated progenitor recruitment and adipogenesis [5,6]; indiscriminate suppression of adipogenesis is therefore not a rational therapeutic objective. Likewise, the AVU model proposes that reproductive signals may reveal or amplify regional susceptibility rather than that systemic estrogen excess universally causes lipedema and provides no rationale for systemic anti-estrogen therapy or hormonal suppression.

If regenerative lock-in exists, therapeutic responsiveness may depend on disease state: dynamically maintained signaling abnormalities might be more reversible than structurally embedded remodeling. The translational value of the AVU framework is therefore not a new treatment algorithm, but a strategy for identifying which abnormalities drive or maintain the pathological tissue state and which interactions must be interrupted to restore regional adipose-tissue homeostasis. The clinical implications, current evidence, and experimental priorites derived vom the AVU framework are summarized in Table 2.

## 8. Alternative Models, Limitations, and Boundaries of the AVU Hypothesis

The integrative capacity of the AVU hypothesis should not be mistaken for evidence that the AVU is the unique or primary site of disease initiation. Most current studies are cross-sectional, involve relatively small cohorts, and examine established disease; temporal sequence, necessity, and sufficiency therefore remain unresolved.

### 8.1. Competing Cellular and Tissue-Level Models

Competing models remain plausible. A primary adipocyte/progenitor defect could initiate regional expansion with vascular, immune, matrix, and lymphatic changes occurring secondarily; a stable cell-autonomous defect sufficient to reproduce the phenotype would reduce the need for an AVU-level explanation. Lipedema-specific studies also support microvascular/perivascular and immune abnormalities [14,15,16], whereas lymphatic and interstitial findings remain heterogeneous [23,24,25,26,27,28,29,30]. ECM and mechanical mechanisms likewise remain plausible [22,49]. Current evidence does not establish temporal primacy for any of these compartments. The AVU model accommodates them as possible entry points but requires reciprocal regional interactions to add explanatory value beyond a single-compartment model.

### 8.2. Systemic, Endocrine, and Genetic Susceptibility

Endocrine and genetic mechanisms remain compatible with the model. A purely systemic hormonal explanation faces the regionality problem, whereas familial clustering and genetic association suggest inherited susceptibility without establishing a single causative gene [31,32]. Genetic or developmental variation could influence progenitor biology, vascular stability, ECM, steroid metabolism, immune regulation, or lymphatic capacity. The AVU is therefore best viewed as a candidate level of regional disease expression and maintenance, not necessarily the deepest etiological level.

### 8.3. Biological Heterogeneity, Obesity, and Diagnostic Uncertainty

Obesity is a major confounder because it frequently coexists with lipedema and shares adipocyte hypertrophy, inflammation, ECM remodeling, and vascular changes [2,3,17]. However, overlap in tissue features does not establish biological equivalence. Obesity-associated adipose dysfunction is strongly linked to impaired adipose expandability, ectopic fat deposition, insulin resistance and cardiometabolic disease [17]. By contrast, women with lipedema have been reported to show greater lower-body adiposity with higher insulin sensitivity than women matched for obesity and whole-body adiposity [44], and another cohort found a lower prevalence of impaired glucose metabolism/insulin resistance than in women with lifestyle-associated overweight/obesity [45]. These data are limited and do not exclude metabolic disease, particularly when obesity coexists. Mechanistic studies should therefore include lipedema with and without obesity, obesity without lipedema, and anatomically matched healthy controls, with rigorous documentation of body composition and metabolic status rather than BMI alone.

Different upstream perturbations may converge on overlapping tissue states, so reproducible AVU patterns or trajectories may ultimately be more informative than a universal biomarker. This possibility requires standardized phenotyping and tissue sampling and should not be used to blur diagnostic boundaries.

### 8.4. Limitations of Current Evidence

The principal limitation is temporal. Most tissue studies examine established disease, so observed endothelial, immune, progenitor, adipocyte, matrix, and lymphatic abnormalities may be initiators, amplifiers, consequences, or adaptations. No study has demonstrated that an AVU-level pathological state persists autonomously after withdrawal of a candidate stimulus. The AVU itself is a proposed spatial and functional construct, and its precise biological boundaries remain to be defined. Heterogeneous diagnostic criteria, small cohorts, variable anatomical sampling, obesity, disease stage, and ex vivo culture conditions further limit comparison across studies.

### 8.5. Boundary Conditions and Falsifiability

A systems model risks accommodating almost any finding after the fact. We therefore define explicit boundaries: the hypothesis does not claim that estrogen is the universal cause, endothelium is always primary, lymphatic dysfunction is necessarily secondary, macrophages universally initiate disease, EVs are sufficient, fibrosis is always late, or all patients follow the same trajectory. Its narrower claim is that a regionally susceptible regenerative niche may provide the tissue-level system in which different upstream susceptibilities converge, interact, and potentially become self-maintaining.

### 8.6. AVU as a Convergence Model

The AVU need not be where lipedema begins. Different upstream routes—genetic/developmental susceptibility, progenitor abnormalities, vascular instability, ECM/mechanical changes, immune dysregulation, endocrine processing, or lymphatic vulnerability—may converge on the same regional tissue system.

The hypothesis should be revised or rejected if regional tissue biology proves irrelevant to disease distribution; affected and spared depots are biologically equivalent; key phenotypes are entirely cell autonomous; cross-compartment interactions are not reproducible; or AVU abnormalities have no relationship to clinical phenotype. It would gain support from reproducible regional multicellular signatures, functional transfer between compartments, persistence after stimulus withdrawal, and reversal after targeted network perturbation.

The contribution of the AVU framework is therefore organizational and experimental: it converts a heterogeneous literature into separable questions about where susceptibility resides, how compartments interact, which abnormalities are causal, and whether a pathological regional state can persist or be reversed.

## 9. Conclusions

Lipedema is a biologically complex disorder of subcutaneous adipose tissue for which no single cellular abnormality or tissue compartment has been established as the universal initiating lesion. Its female predominance, regional distribution, and reported abnormalities across adipogenic, vascular, immune, matrix, interstitial, and lymphatic compartments argue for a model that can integrate systemic physiology with local tissue susceptibility.

Current evidence does not establish the AVU as the primary site of initiation or demonstrate regenerative lock-in. Lipedema-specific observations support abnormalities in several AVU compartments, whereas general adipose-tissue studies provide mechanistic plausibility for reciprocal niche interactions; the proposed causal links remain hypotheses until tested. The model is valuable only if its predictions survive testing: affected and spared depots should show specific multicellular differences, endocrine responses should be context-dependent, intercompartmental interactions should be functionally demonstrable, regional boundaries should have biological correlates, and any proposed self-maintaining state should show measurable persistence and reversibility.

The AVU hypothesis therefore reframes lipedema as a testable problem of regional regenerative homeostasis. Paired-depot studies, spatial profiling, multicellular reconstruction, causal perturbation, and longitudinal investigation can determine whether AVU dysregulation is a causal organizing principle of lipedema biology or a descriptive consequence of established disease.

## Figures and Tables

**Table 1:** Experimental roadmap and falsifiable outcomes for the AVU hypothesis.

Question	Specific Prediction	Preferred Approach	Finding That Would Weaken the AVU Model
Where is susceptibility encoded?	Affected depots show a coordinated AVU signature beyond adipocyte size/BMI.	Paired affected-spared sampling; single-cell/spatial profiling; obesity controls.	No reproducible affected-spared difference or differences explained by obesity alone.
Is endocrine responsiveness regional?	Physiological reproductive signals produce depot-specific responses.	Matched ex vivo tissue/organoid stimulation across endocrine conditions.	Equivalent responses across affected and spared depots.
Does multicellular context matter?	Disease-associated behavior is stronger in reconstructed multicellular systems than in monoculture.	Co-culture/organoid systems with endothelial, mural, ASPC, immune and ECM components.	Stable phenotype reproduced entirely by isolated cells.
Do EVs transmit phenotype?	Defined lipedema-associated EV cargo produces recipient-cell effects.	EV transfer plus cargo-specific gain/loss experiments.	No reproducible transfer or cargo dependence.
Is regenerative lock-in present?	Disease-associated signaling persists after stimulus withdrawal and is reversible by network perturbation.	Time-resolved withdrawal, perturbation, and rescue experiments.	Rapid complete normalization after withdrawal.
Does AVU biology matter clinically?	AVU states correlate with distribution, symptoms, progression, or treatment response.	Prospective longitudinal cohorts with standardized phenotyping and tissue sampling.	AVU states track only BMI, tissue volume, or conventional stage without added information.

**Table 2:** Clinical implications, current evidence and experimental priorities derived from the AVU framework.

Clinical/Research Domain	Current Evidence or Practice	AVU Interpretation	Key Test/Future Direction
Conservative management	Compression, physical activity, rehabilitation and weight management target symptoms, function, fluid handling and comorbidity [2,3].	Phenotype-directed; benefit does not identify the initiating mechanism.	Relate clinical response to regional AVU biomarkers rather than assuming mechanism from symptomatic improvement.
Coexisting obesity/metabolic risk	Obesity can coexist with lipedema and independently increase metabolic risk; lipedema cohorts may nevertheless show comparatively preserved metabolic profiles [44,45].	Metabolic burden and regional lipedema biology should be separated analytically.	Stratify by obesity and metabolic status; compare with obesity-without-lipedema controls.
Reduction surgery	Removes affected subcutaneous tissue and improves symptoms in selected patients [46,48].	May reduce tissue burden and disrupt multiple AVU compartments; disease modification is unproven.	Longitudinal paired tissue/clinical profiling before and after surgery.
Progenitor-vascular/immune-stromal signaling	Candidate interactions are biologically plausible; causal lipedema-specific nodes are not established.	Potential future mechanism-directed targets.	Co-culture, perturbation and rescue experiments demonstrating necessity and sufficiency.
EV-mediated communication	Lipedema SVF EVs show altered miRNA cargo [13].	Possible route of intercellular amplification.	Cargo-specific transfer and gain/loss experiments in recipient AVU cell types.
ECM/fibrosis and regenerative lock-in	Stage-associated fibrosis is observed [22]; self-maintaining AVU signaling after stimulus withdrawal is unproven.	Structural stabilization must be distinguished from active network persistence.	Stimulus-withdrawal experiments and targeted network disruption in multicellular models.

## Data Availability

No new data were created or analyzed in this study. Data sharing is not applicable to this article.
